# Bisphenol A at a human exposed level can promote epithelial‐mesenchymal transition in papillary thyroid carcinoma harbouring *BRAF* ^V600E^ mutation

**DOI:** 10.1111/jcmm.16279

**Published:** 2021-01-19

**Authors:** Liuli Li, Hao Li, Jun Zhang, Xin Gao, Hao Jin, Renqi Liu, Zhen Zhang, Xuan Zhang, Xichang Wang, Peng Qu, Yuejiao Zhao, Xiaobo Lu

**Affiliations:** ^1^ Department of Toxicology School of Public health China Medical University Shenyang China; ^2^ Department of Oromaxillofacial–Head and Neck Surgery Department of Oral and Maxillofacial Surgery School of Stomatology China Medical University Shenyang China; ^3^ Department of head and Neck Surgery Cancer Hospital of China Medical University/Liaoning Cancer Hospital & Institute Shenyang China; ^4^ Jin Zhou Center for Disease Control and Prevention Jinzhou China

**Keywords:** Bisphenol A, *BRAF*^V600E^ mutation, epithelial‐mesenchymal transition, ERK‐Cox2, Papillary thyroid carcinoma

## Abstract

Bisphenol A (BPA), a ubiquitous endocrine‐disrupting chemical, alters the function of endocrine system and enhances the susceptibility to tumorigenesis in several hormone‐dependent tumours as thyroid carcinoma. About 50% of papillary thyroid cancers (PTC), the most common type of thyroid malignancy, harbours the *BRAF* ^V600E^ mutation. This study aimed to investigate a potential combined effect of BPA exposure and *BRAF* ^V600E^ mutation on epithelial‐mesenchymal transition (EMT) in PTC. Firstly, the level of BPA in plasma, the evaluation of BRAF^V600E^ mutation and the level of EMT‐related proteins in PTC samples were individually determined. Additionally, the migration, invasion, colony formation capacity and the expression of EMT‐related proteins after exposure to BPA were precisely analysed in vitro thyroid cells genetically modified by the introduction of *BRAF* ^V600E^ mutation. Moreover, ERK‐Cox2 signalling pathway was also introduced to explore the possible mechanism in PTC development. As expected, whether the clinical investigation or cultured thyroid cells demonstrated that BPA at a concentration compatible with human exposed levels (10^‐7^ M) synergized with the *BRAF* ^V600E^ mutation promoted EMT via the activation of ERK‐Cox2 signalling pathway. Our findings offer some evidence that BPA as an environmental risk factor can facilitate the progression of PTC harbouring *BRAF* ^V600E^ mutation.

## 1 INTRODUCTION

Thyroid carcinoma has been appreciably increasing worldwide in recent decades, which gradually becomes the most common solid malignant tumour of the endocrine system and is highly prevalent in middle‐aged people.^1^ Generally speaking, papillary thyroid carcinoma (PTC), the most common thyroid malignancy, accounts for over 80% of all cases and is usually associated with favourable survival.^2^ However, recurrence, metastases and cancer death may occur in 10%‐15% of patients with more aggressive types, such as epithelial cell‐derived PTC.^3^ Therefore, studies on the detailed mechanisms underlying PTC metastasis is of significance in improving our understanding of the pathogenesis process and identifying valid, targeted treatments.

Epithelial‐mesenchymal transition (EMT) is a biological process that epithelial cells lose their junctions and apical‐basal polarity, and acquire migratory and invasive capabilities.^4^ In the context of neoplasias, EMT confers on cancer cells increased tumour‐initiating and metastatic potential,^5^ in particular, epithelial‐derived malignant cells.^6^ The cells consequently acquire a spindle‐shaped mesenchymal morphology and express markers associated with the mesenchymal cell state, notably neural cadherin (N‐cadherin) and matrix metallo proteinase‐9 (MMP‐9), while the expression of E‐cadherin and certain cytokeratins is typically lost.^7^, ^8^

Genetic abnormalities and epigenetic alterations have been recognized as a crucial driver in EMT. Remarkably, *BRAF* ^V600E^ mutation, the transformation of valine (V) to glutamic acid (E) at amino acid 600 (pV600E) resulting from T‐to‐A (c1799T > A) substitution at nucleotide 1799, has been identified as a targetable, oncogenic mutation in many cancers, present in some 30%‐70% of PTC with pronounced transcriptomic and prognostic consequence.^9^ Mechanistically, the mutation constitutively activates the BRAF kinase in the MAPK pathway to promote aggressiveness and contribute to a worse prognosis, advanced stage, lymph node metastasis as well as the resistance to traditional radiation therapy of PTC.

Bisphenol A (BPA) as a synthetic ubiquitous endocrine‐disrupting chemical (EDC) is used greatly in the manufacture of plastic products.^10^, ^11^, ^12^ The growing epidemiological studies indicate that BPA has been associated mainly with hormone‐sensitive cancers including thyroid cancers as it interacts with oestrogen receptors and acts as an agonist or antagonist via oestrogen receptor‐dependent signalling pathways. For example, Zhou Z’s study indicated that the BPA level in urine of PTC patients was higher than the healthy control, which reminded that BPA exposure may potentially promote the pathogenesis and progression of PTC^12^; Soto's studies suggested the increasing incidence of thyroid carcinoma was associated with BPA exposure in almost all age groups.^13^ Recent evidence has highlighted that BPA has an increasingly crucial role in EMT. For instance, Zhai's study indicated that BPA regulated snail‐mediated EMT in hemangioma cells, consistently with Oral's investigation in ovarian cells and Kim's research in human breast cancer cells MCF‐7 CV.^14^, ^15^, ^16^

Despite inroads that have been made in identifying *BRAF* ^V600E^ mutation's potential role in the EMT process of PTC, the joint effect of BPA exposure on EMT induced by *BRAF* ^V600E^ mutation remains uncertain. Accordingly, a clinical investigation of PTC patients and an in vitro genetically emerging thyroid cell model was performed in the present study to assess whether BPA exposure especially at a human exposed level combined with *BRAF* ^V600E^ mutation could promote the EMT process and further enhance migration and invasion of thyroid cells. Moreover, ERK‐Cox2 as an activated signalling pathway was also introduced to explore the possible mechanism of thyroid carcinogenesis. Hopefully, our current study may contribute to understanding scientific significance for early prevention of thyroid carcinoma related to endocrine‐disrupting chemicals exposure.

## 2 MATERIALS AND METHODS

### 2.1 Materials pre‐treatment

In order to reduce the interference of environmental BPA pollution in our experiment, all the consumables (including blood collection tubes, containers and pipettes) were made of glass products in the subject process. The washed glassware was first of all soaked in potassium dichromate solution for 24 hours, afterwards rinsed with ultrapure water and then rinsed with 75% ethanol solution, at last, dried in a dry box before use. The plastic consumables used in vitro were made of general purpose polystyrene without BPA.

### 2.2 Clinical samples

The study population consisted of 45 PTC patients in Liaoning Cancer Hospital & Institute (Liaoning Province, China) and 50 healthy Chinese Han participants as control were collected from June 2017 to November 2018. The protocol and consent form were approved by the Institutional Review Board of China Medical University prior to the study. All activities involving human subjects were done in full compliance with government policies and the Helsinki Declaration. After the study procedures were explained and all questions were answered, subjects signed informed consent forms. Each participant donated 5 ml venous blood and all PTC patients provided intraoperative fresh carcinoma tissues. Meanwhile, their demographic data were recorded in questionnaires.

### 2.3 Detection of BPA level in plasma

Human BPA ELISA kit, purchased from Shanghai Enzyme‐linked Biotechnology Company, was used to measure BPA content in plasma of the study subjects. Used suited EDTA as an anticoagulant, mixed 15 minute, centrifugated 20 minute at the speed of  1000 g and collected supernatant. Then, the experimental procedure and formulas for the calculation strictly followed the kit instructions.

### 2.4 Evaluation of *BRAF* ^V600E^ mutation in tumour

Immunohistochemistry was performed as previously described to detect the *BRAF* ^V600E^ mutation.^17^ PTC tissue samples were sliced into 4‐μm sections and incubated overnight at 4°C with the addition of a commercially available *BRAF* ^V600E^ mutation‐specific antibody (dilution 1:100, clone VE1; Spring Bioscience, Pleasanton, CA, USA). Further, the sections were incubated with biotin‐labelled secondary goat anti‐rabbit immunoglobulin G (IgG) at 37°C for 20 minutes, stained with diaminobenzidine and counterstained with haematoxylin. The negative controls were performed by omitting the primary antibody. The *BRAF* ^V600E^ mutation was evaluated by two experienced pathologists (DES) with the blind method. Diffuse homogeneous cytoplasmic staining in cells was considered as a positive *BRAF* ^V600E^ mutation, while non‐specific staining of colloids and equivocally weak or focal cytoplasmic staining was considered as *BRAF* ^V600E^ wild‐type.

### 2.5 Immunohistochemical staining

The procedure of immunohistochemical staining for E‐cadherin, N‐cadherin and MMP‐9 was the same as the previous description. The slides were incubated with the addition of the following primary antibodies: E‐cadherin (dilution 1:400, #3195), N‐cadherin (dilution 1:125, #13116) and MMP‐9 (dilution 1:325, #13667). These antibodies were all purchased from Cell Signaling Technology (CST, USA). Finally, the slides were scanned and images were captured in 5 randomly selected visual fields for analysis under a Digital Pathology Scanner (Aperio CS2, Leica Biosystems, USA).

The intensity of the dye colour was graded as 0 (no colour), 1 (light yellow), 2 (light brown), or 3 (brown), and the number of positive cells was graded as 0 (<5%), 1 (5%‐25%), 2 (25%‐50%), 3 (51%‐75%) or 4 (>75%). The two grades were multiplied together and specimens were assigned to one of 4 levels: 0‐1 score (−), 2‐4 scores (+), 5‐8 scores (++), more than 9 scores (+++).^18^

### 2.6 Cell culture

Immortalized human thyroid follicular epithelial cell line Nthy‐ori 3‐1 (No.BNCC340487, *BRAF* wild‐type) was purchased from Bena Culture Collection (Suzhou, China). The cells were grown as monolayers in no phenol red DMEM**/**F‐12 (Gibco, Invitrogen Corporation, USA) supplemented with 10% foetal bovine serum (treated with 5% activated carbon before use) and 10 IU/ml penicillin**/**streptomycin and maintained at 37°C, 5% CO_2_ in a humidified incubator.

### 2.7 Plasmids transfection

The pIRESpuro3‐EGFP‐Flag‐6 × His‐*BRAF*‐T1799A plasmid and the control plasmid pIRESpuro3‐EGFP‐Flag‐6 × His (OBio, Shanghai, China) were transfected by Lipo8000™ reagent (Beyotime, China) according to the manufacturer's instructions. After 48 hours transfection, the medium was replaced with different concentrations of BPA‐containing complete medium and the cells were further detected for the following biological function tests.

### 2.8 Preparation of BPA‐containing culture medium

BPA (Chengdu Aikeda Chemical Reagent Co., Ltd., China) was dissolved in dimethyl sulfoxide (DMSO, Sigma‐Aldrich, USA) to form a 1 M stock solution stored at −20°C. The stock solution was diluted with complete medium to obtain a drug‐containing culture medium with a final BPA concentration of 10^‐3^ to 10^‐10^ M for cell treatment. The control group was treated with a complete culture medium containing 0.1% DMSO.

### 2.9 Cell proliferation assay

Cells were seeded in 96‐well plates at a density of 3000 cells per well. After 24 hours incubation, the cells were treated with different concentrations of BPA for 24, 48 or 72 hours. Then 20 μL MTS was added and cells were incubated for 2h to measure the cell viability with a microplate reader (EON, BioTek, USA).

### 2.10 Scratch wound assay

Cells were seeded in a 6‐well plate and transfected when the cell fusion rate reached 70%‐80%. After 48 hours of cell transfection, scratches were made. After treatment with BPA at different concentration, the wound healing of cells at the same location was observed with a digital microscope (EVOS XL Core, Thermo Fisher Scientific, USA) and the migrated rate of the scratch wound was evaluated using Image J software (National Institutes of Health, USA).

### 2.11 Cell migration and invasion assays

Transwell chambers were put into 24‐well plates, and the apical chamber of the basement membrane was coated with Matrigel diluent (Corning, USA). The cells (3 × 10^4^ cells**/**insert for migration assay; 5.5 × 10^4^ cells**/**insert for invasion assay), transfected different plasmids for 48h, were suspended in 150 μL of serum‐free medium and seeded onto the upper chamber, while the lower chamber was loaded with 600 μL complete medium containing different concentrations of BPA. After 24 hours of incubation, the non‐migrating or non‐invading cells were removed from the upper surface of the membrane by gently scrubbing with the cotton‐tipped swab. Cells on the lower surface of the membrane were fixed with methanol and then stained with 0.2% crystal violet followed by two washes with PBS. Thereafter, cells from five randomly selected microscopic fields were photographed and counted (EVOS XL Core, Thermo Fisher Scientific, USA).

### 2.12 Colony formation assay

Cells were incubated in 6‐well plates for 12 hours, then transfected with the corresponding vectors for 48 hours. Afterwards, 2000 cells per well were seeded in 12‐well plates and exposed to different concentrations of BPA. After 7 days, colonies were fixed with 4% paraformaldehyde and stained using 0.2% crystal violet. Finally, colony numbers were counted and analysed using the light microscope (Model 200‐BFFL‐S, Nexcelom Bioscience, USA).

### 2.13 Total RNA extraction and quantitative real‐time PCR

Total RNA was extracted from thyroid carcinoma tissues with Trizol reagent (Invitrogen Inc, Burlington, ON, Canada). One microgram of total RNA was converted to cDNA and then was amplified in duplicate using SYBR Premix Ex Taq II (Takara, Dalian, China) and a Light Cycler 480 II (Roche, Switzerland) PCR detection system. The relative quantification of mRNA levels was calculated by the ln2^−ΔCt^ method, normalized to *GAPDH*. The primer sequences are shown in Table S1.

### 2.14 Western blot analysis

Western blot analysis was performed as described previously with the following antibodies: BRAF (1:1000, #9433), ERK (1:1000, #4695), p‐ERK (1:2000, #4370), Cox2 (1:1000, #12282), E‐cadherin (1:1000, #3195), N‐cadherin (1:1000, #13116), MMP‐9 (1:1000, #13667) and GAPDH (1:1500, #5174) and secondary goat anti‐rabbit immunoglobulin G (ab6721, 1:20 000, Abcam Inc). All primary antibodies were purchased from Cell Signaling Technology (CST, USA). The grey value of protein expression was measured with the NIH software Image J (National Institutes of Health, USA).^19^

### 2.15 Statistical analysis

Data statistical analysis was carried out using the SPSS 20.0 software. The enumeration data were expressed in terms of frequency and percentage and measurement data was expressed as mean ± standard deviation (SD). Comparisons between two groups were determined by Student's t test. Multiple‐group comparisons were performed by one‐way ANOVA and LSD multiple comparison tests. Each independent experiment performed in triplicate and represented as ‘n = 3’, and *P* < .05 was considered as statistically significant.

## 3 RESULTS

### 3.1 Quantitation of BPA exposure in plasma

The demographic data of study subjects were present in supplementary materials (Table S2). BPA inter exposure in plasma of study subjects was at 10^‐7^ M, a common human exposed level, while no significant difference was found between healthy controls and PTC patients. Subsequently, stratified analysis by age, gender, smoking habits and alcohol drinking status demonstrated no statistical difference as well (Table 1).

**TABLE 1:** Comparison of plasma BPA levels between healthy controls and PTC patients

Variables	Plasma BPA levels (10^‐7^ M)		*P* Value
	Healthy controls(n)	PTC patients(n)	
Total	3.02 ± 0.29(50)	2.87 ± 0.53(45)	
Age(years)			
<50	3.05 ± 0.35(24)	2.90 ± 0.61(24)	0.096
≥50	2.95 ± 0.18(26)	2.85 ± 0.42(21)	0.295
Sex			0.274
Female	3.00 ± 0.32(39)	2.86 ± 0.56(36)	
Male	3.07 ± 0.11(11)	2.91 ± 0.39(9)	0.188
Smoking			0.203
Yes	3.00 ± 0.19(12)	2.84 ± 0.29(16)	
No	3.03 ± 0.32(38)	3.05 ± 0.01(29)	0.163
Drinking			0.652
Yes	3.09 ± 0.11(5)	3.04 ± 0.16(10)	
No	3.01 ± 0.30(45)	2.82 ± 0.45(35)	0.662

### 3.2 *BRAF* ^V600E^ **mutation in PTC tissue**

According to the result of immunohistochemical staining with the anti‐*BRAF* ^V600E^ VE1 in tumour tissues from 45 PTC patients, 57.8% of the patients (26/45) were concluded to harbour the *BRAF* ^V600E^ mutation. In detail, the diffused brownish yellow granules in the cytoplasm represented for typical positive *BRAF* ^V600E^ mutation (Figure S1).

### 3.3 *BRAF* ^V600E^ **mutation promoted EMT in PTC**

**FIGURE 1:**
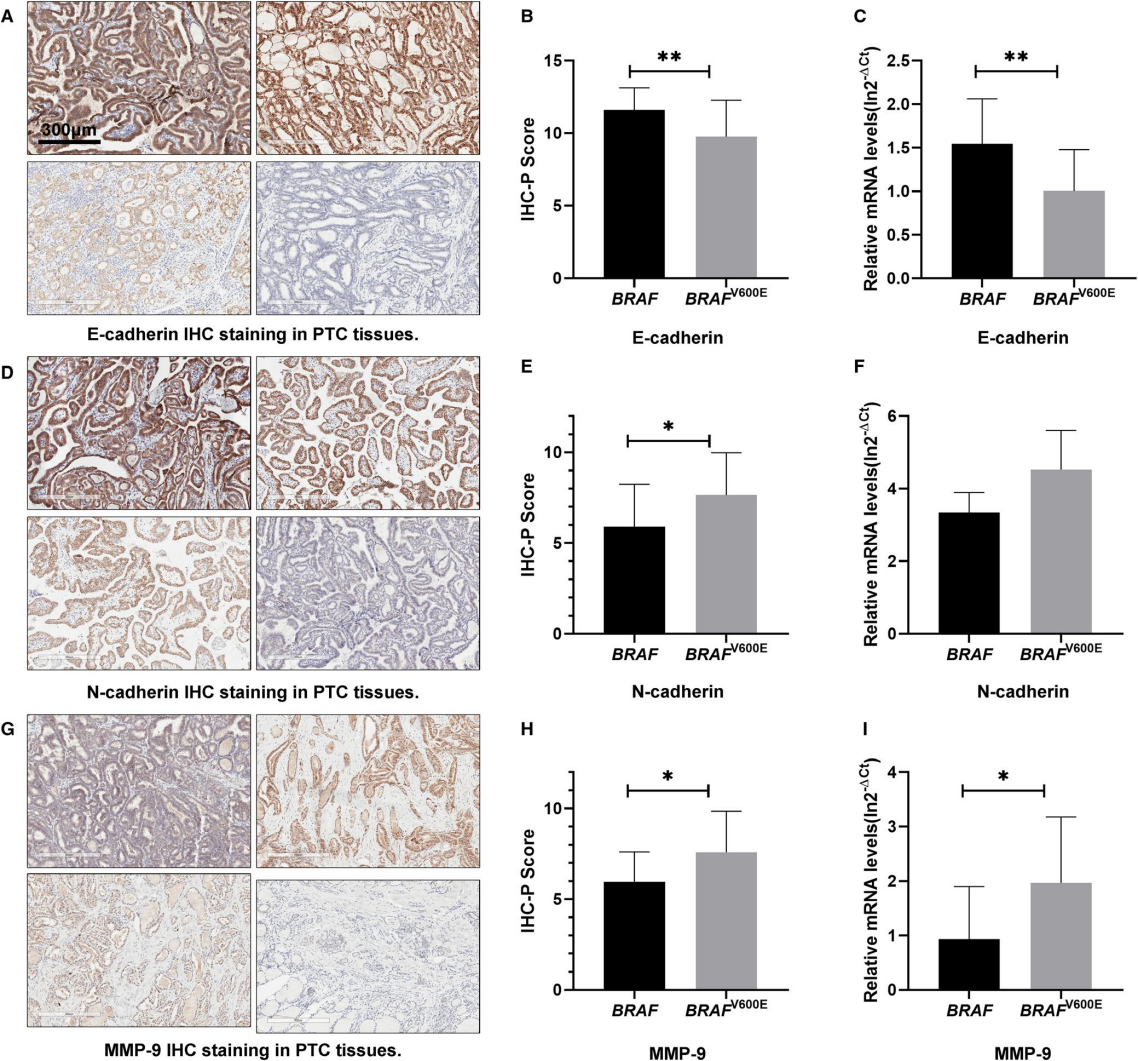
*BRAF* ^V600E^ mutation‐induced EMT in PTC patients. (A, D and G) Typical images of PTC tissue with strong positive, positive, weak positive and negative staining of E‐cadherin (A), N‐cadherin (D) and MMP‐9 (G) in proper order. (B, E and H) Representative for the protein expression of E‐cadherin, N‐cadherin and MMP‐9, respectively, in the carcinoma tissues of PTC patients with wide‐type or mutated *BRAF* ^V600E^. (C, F and I) Representative for the mRNA expression of E‐cadherin, N‐cadherin and MMP‐9, respectively, in the carcinoma tissues of PTC patients with wide‐type or mutated *BRAF* ^V600E^. *P < .05, **P < .01

The IHC‐P Scores (IHS) of EMT‐related proteins were shown in Figure 1. We found that the epithelial cell marker E‐cadherin expression in *BRAF* ^V600E^ mutant tissues was significantly repressed compared with the wild‐type ones (Figure 1B), whereas N‐cadherin and MMP‐9 protein expression was significantly up‐regulated in *BRAF* ^V600E^ mutated samples (Figure 1E and H). Consistently, transcriptional repression of E‐cadherin but activation of N‐cadherin and MMP‐9 was observed in *BRAF* ^V600E^ mutated samples (Figure 1C, F and I). The above results suggest that the *BRAF* ^V600E^ mutant carcinoma is more prone to promote EMT than the wild‐type ones.

### 3.4 BPA exposure enhanced EMT in PTC harbouring *BRAF* ^V600E^ mutation

**FIGURE 2:**
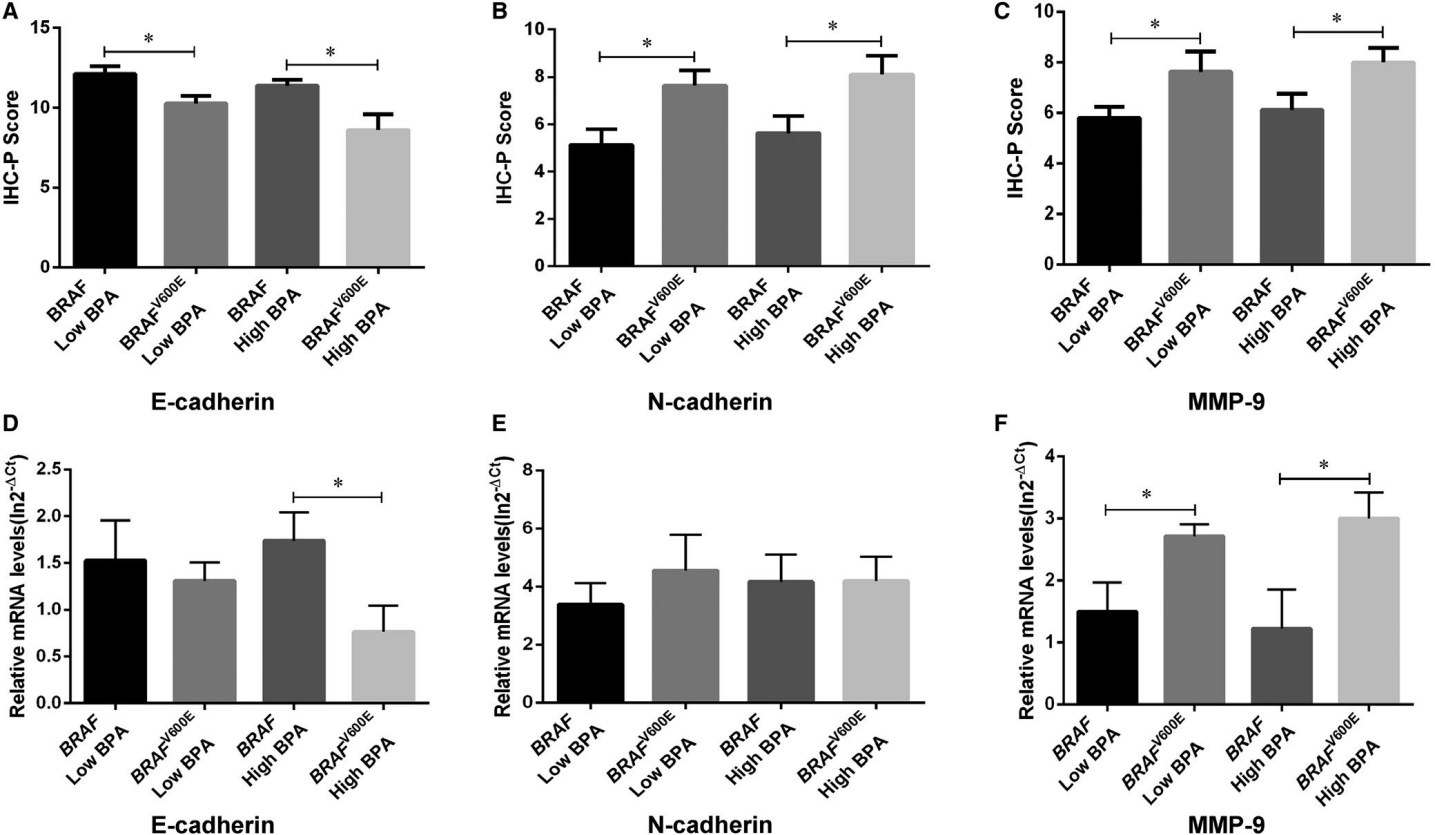
BPA exposure enhanced EMT in PTC patients harbouring *BRAF* ^V600E^ mutation. (A‐C) Representative for the IHC‐P Score of E‐cadherin, N‐cadherin and MMP‐9. (D‐F) Representative for the mRNA expression of E‐cadherin, N‐cadherin and MMP‐9. *P < .05

A total of 45 PTC patients were divided into low BPA exposure and high BPA exposure groups according to the median BPA exposure level in plasma, and the IHS was further analysed in combination with the varied *BRAF* genotypes. In the BPA low‐exposure group, E‐cadherin protein expression was decreased (Figure 2A), while N‐cadherin (Figure 2B) and MMP‐9 (Figure 2C) were increased in PTC patients with *BRAF* ^V600E^ mutation compared with wild‐type ones. Meanwhile, in the BPA high‐exposure group, the above proteins showed a similar trend to BPA low exposure. Remarkably, E‐cadherin mRNA expression showed significant repression in ones harbouring *BRAF* ^V600E^ mutation combined exposed to high BPA concentration, but such a trend was not observed in the low BPA exposure group (Figure 2D); Meanwhile, high BPA concentration might induce the repression of E‐cadherin but the activation of N‐cadherin and MMP‐9 protein expression in *BRAF* ^V600E^ mutated tissues in spite of no statistical significance (Figure 2A‐2C). The above results indicated that BPA exposure might promote EMT in PTC harbouring *BRAF* ^V600E^ mutation.

### 3.5 Establishment of the *BRAF* ^V600E^ overexpressed cell model

**FIGURE 3:**
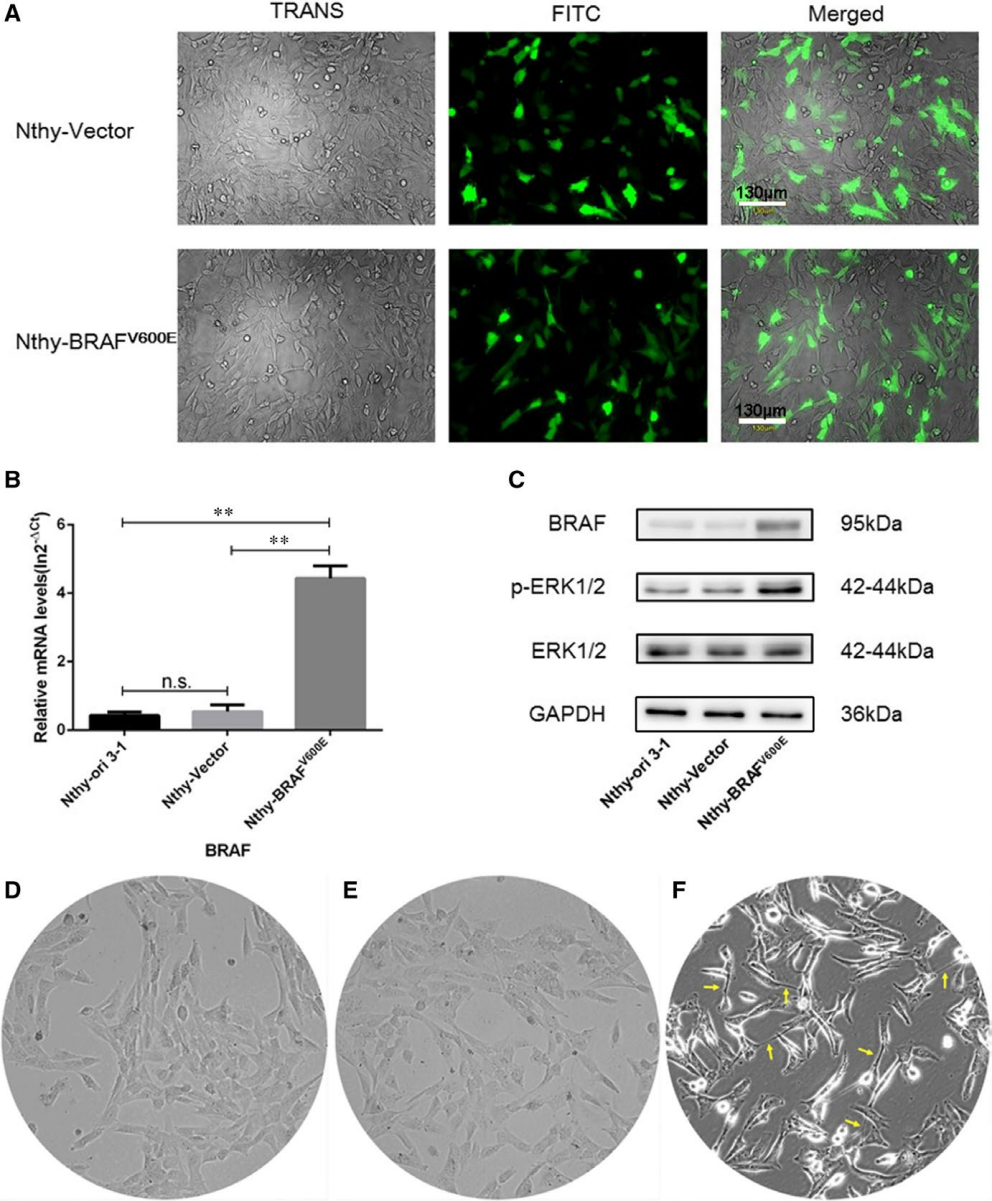
Establishment of the *BRAF* ^V600E^ overexpressed cell model. (A) Typical fluorescence images of Nthy‐ori 3‐1 cells after 48h's transfection with different plasmids. (B) The mRNA level of *BRAF* gene after 48h's transfection in Nthy‐ori 3‐1 cells. (C) Protein expression levels of BRAF, p‐ERK, ERK after 48h's transfection in Nthy‐ori 3‐1 cells, normalized to GAPDH. (D‐F) Typical images of parental Nthy‐ori 3‐1 cells (D) and cells transfected with control (E) or *BRAF* ^V600E^ mutated plasmid (F). The yellow arrow in the figure (F) marked the spindle transformed shape cells. **P < .01

The transfection efficiency in the present experiment was up to 60%‐75% after 48h's transfection (Figure 3A). Subsequently, we confirmed that compared with the parental and empty vector‐transfected Nthy‐ori 3‐1 cell, the introduction of *BRAF* ^V600E^‐mutated vector significantly increased the expression of BRAF (Figure 3B). In addition, activation of BRAF‐ERK signalling pathway in Nthy‐*BRAF* ^V600E^ cells (Figure 3C), which was evidenced by the increased protein levels of BRAF and p‐ERK, indicated that the *BRAF* ^V600E^ overexpressed thyroid cell model was successfully established. Remarkably, Nthy‐*BRAF* ^V600E^ cells had a spindle transformed shape other than the Nthy‐Vector cell after 48h's transfection, as shown in Figure 3D‐3F.

### 3.6 BPA exposure augmented *BRAF* ^V600E^‐induced migration/invasion in thyroid cells

To determine the cytotoxicity of BPA, Nthy‐ori 3‐1 cell line was treated with different concentrations of BPA ranging from 10^‐10^ to 10^‐3^ M for 24, 48 and 72hrs, and then cell viability was assessed by MTS kit assay. Our results revealed that BPA treatment promoted cell growth at the concentration of 10^‐10^ to 10^‐4^ M while inhibited cell growth at 10^‐3^ M, where the most significant proliferation was observed at 10^‐7^ M (Supplementary Figure 2A and 2B). Furthermore, the cells were treated by BPA (10^‐4^ to 10^‐3^ M) to obtained the inhibition curve (Figure 2C), where 3.33 × 10^‐4^ M was determined as IC_15_ of BPA. Thereby, we considered 3.33 × 10^‐4^ M and 10^‐7^ M as the candidate doses in the subsequent in vitro experiments.

**FIGURE 4:**
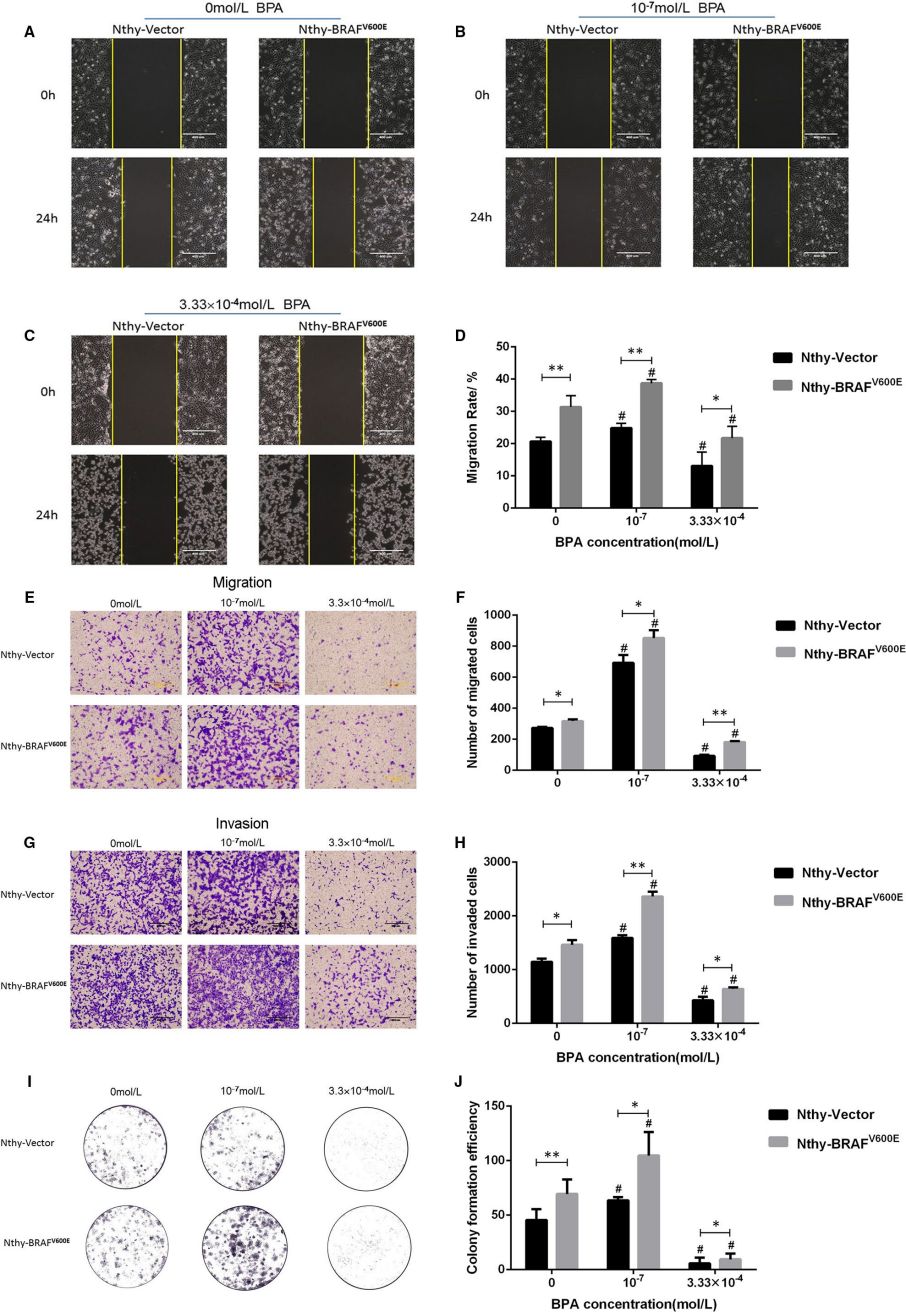
BPA exposure augmented *BRAF* ^V600E^‐induced migration/invasion of Nthy‐ori 3‐1 cells. (A‐C) Typical images of the scratch assay in Nthy‐ori 3‐1 cells transfected with different plasmids combined exposure to BPA at the concentrations of 0, 10^‐7^ M and 3.33 × 10^‐4^ M, respectively. Scale bars are 400 µm. (D) Representative for the migration rate of scratch assay in transfected cells treated with different concentrations of BPA. (E) Typical images of Transwell migration experiment in transfected cells treated with different concentrations of BPA. Scale bars are 400µm. (F) Representative for the number of migrated cells in transfected cells treated with different concentrations of BPA. (G) Typical images of Transwell invasion assay in transfected cells treated with different concentrations of BPA. (H) Representative for the number of invading cells in transfected cells treated with different concentrations of BPA. (I) Typical images of colony‐forming assay in transfected cells treated with different concentrations of BPA. (J) Qualification of the above colony‐forming assay. Data presented as mean ± SD. *P < .05, **P < .01, ^#^ *P* < .05 *vs*. the control group

Subsequently, scratch wound assay, Transwell migration and Matrigel invasion assays were used to study the effect of *BRAF* ^V600E^ mutation and BPA exposure on the malignant phenotypes of thyroid cells. Compared with Nthy‐Vector cells, the mobility of Nthy‐*BRAF* ^V600E^ cells significantly increased under the treatment of 0, 10^‐7^ M and 3.33 × 10^‐4^ M BPA (Figure 4A–4D), indicating that *BRAF* ^V600E^ mutation can enhance the cells migration ability. Compared with the control, Nthy‐Vector and Nthy‐*BRAF* ^V600E^ cells exposed to 10^‐7^ M BPA migrated to the scratch and healed the wound more quickly (Figure 4B), while cell migration rate was markedly declined after 3.33 × 10^‐4^ M BPA treatment (Figure 4C). Accordingly, we concluded that 10^‐7^ M BPA could promote the migration of Nthy‐ori 3‐1 cell, while 3.33 × 10^‐4^ M BPA had a contrary effect.

Consistently with the above results of scratch test, *BRAF* ^V600E^ mutation and 10^‐7^ M BPA exposure augmented the migration of Nthy‐ori 3‐1 cells, conversely, the migration was suppressed after 3.33 × 10^‐4^ M BPA exposure (Figure 4E and 4F). Notably, cells with *BRAF* ^V600E^ mutation combined 10^‐7^ M BPA exposure undergoing high migration (Figure 4E and 4F).

The results Matrigel invasion assays revealed that the *BRAF* ^V600E^ mutation significantly enhanced the invasiveness of thyroid cells (Figure 4G and 4H). Hereafter, the cell treated by 10^‐7^ M BPA had stronger invasive ability than the control, while 3.33 × 10^‐4^ M BPA treatment markedly reduced the number of invaded cells (Figure 4G and 4H), suggesting that 10^‐7^ M BPA induced the Nthy‐ori 3‐1 cells’ invasiveness, while 3.33 × 10^‐4^ M BPA had an opposite effect. Similarly, cells with *BRAF* ^V600E^ mutation combined 10^‐7^ M BPA exposure undergoing highly invasive mesenchymal transformation (Figure 4G and 4H).

The plate colony formation assay was carried out to evaluate the cell population dependence and proliferative capacity. The results showed that compared with Nthy‐Vector cells, the clone number of Nthy‐*BRAF* ^V600E^ cells significantly increased at 0, 10^‐7^ M and 3.33 × 10^‐4^ M BPA treatment, indicating that *BRAF* ^V600E^ mutation could enhance cell plate colony formation (Figure 4I and 4J). Compared with the control group, the significant increasing clone number in 10^‐7^ M BPA group but the reduced clone number in Nthy‐Vector and Nthy‐*BRAF* ^V600E^ cells after 3.33 × 10^‐4^ M BPA exposure were observed, reminding that cell colony formation could be promoted by 10^‐7^ M but be inhibited by 3.33 × 10^‐4^ M BPA exposure (Figure 4I and 4J).

### 3.7 BPA exposure accelerated *BRAF* ^V600E^‐induced EMT via activating the ERK‐Cox2 signalling pathway

**FIGURE 5:**
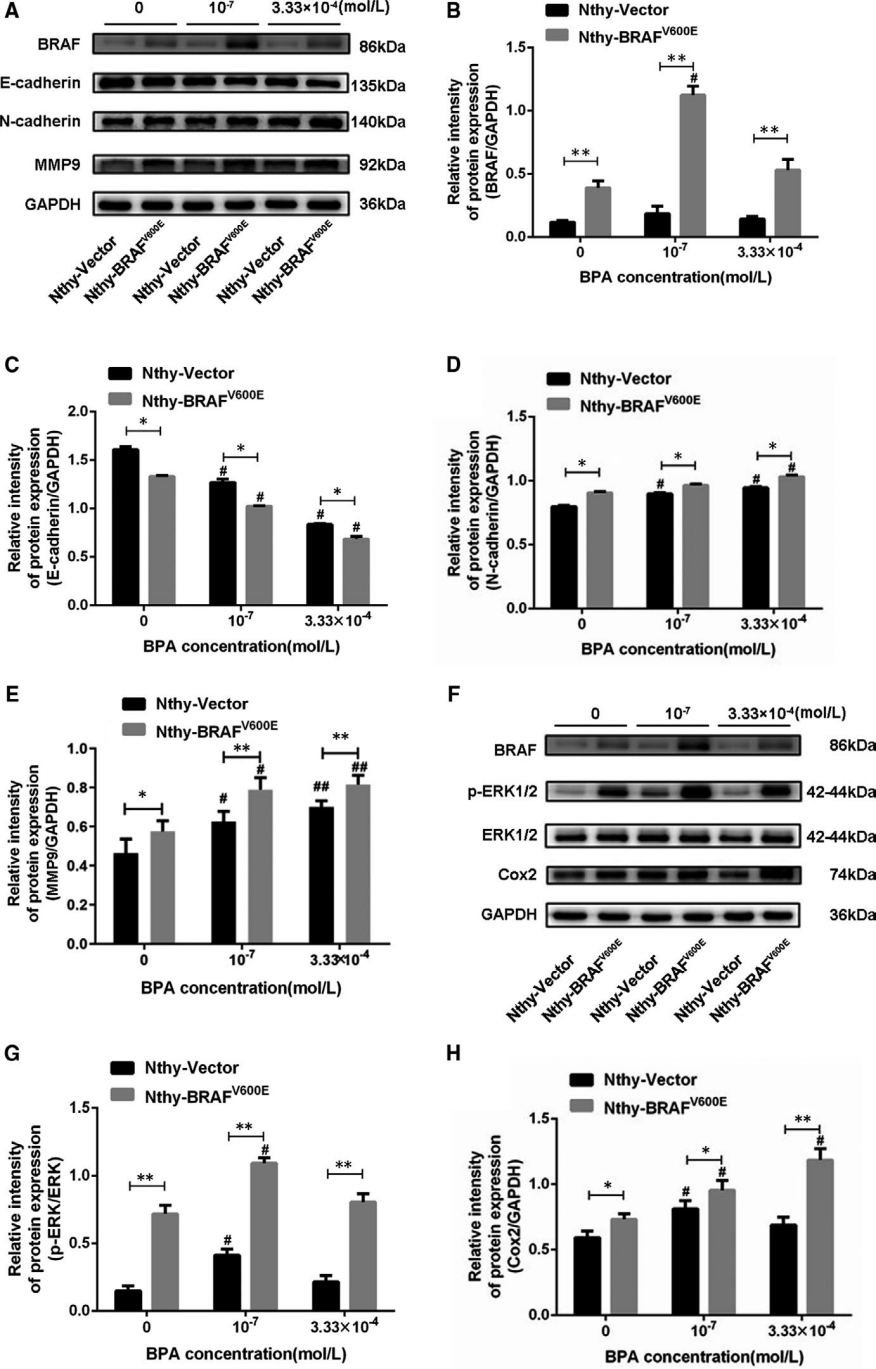
BPA exposure accelerated *BRAF* ^V600E^‐induced EMT of Nthy‐ori 3‐1 cell via activating the ERK‐Cox2 signalling pathway. (A) The protein expressions of BRAF, MMP‐9, E‐cadherin and N‐cadherin proteins in transfected cells treated with different concentrations of BPA. (B‐E) Relative intensity of BRAF, E‐cadherin, N‐cadherin and MMP‐9 protein expression. (F) The protein expression of BRAF p‐ERK, ERK and Cox2 in transfected cells treated with different concentrations of BPA. (G‐H) Relative intensity of p‐ERK/ERK and Cox2 protein expression. Data presented as mean ± SD. *P < .05, **P < .01; ^#^ *P* < .05, ^##^ *P* < .01 *vs*. the control group

Compared with the control group, 10^‐7^ M BPA treatment can significantly promote EMT in Nthy‐Vector and Nthy‐*BRAF* ^V600E^ cells, evidenced by the gain of mesenchymal markers N‐cadherin and MMP9 or the loss of epithelial marker E‐cadherin (Figure 5A‐5E), whereas 3.33 × 10^‐4^ M BPA treatment also activated the expression of EMT‐related proteins (Figure 5A‐5E). It suggested that the inhibitory effect of 3.33 × 10^‐4^ M BPA on the migration, invasion and clonality of transfected cells might be mainly ascribed to the inhibitory effects of high dose of BPA on cells proliferation, rather than the EMT process. Remarkably, *BRAF* ^V600E^ mutation combined 10^‐7^ M BPA exposure significantly promoted the expression of EMT markers (E‐cadherin reduction and N‐ cadherin, MMP‐9 induction) in thyroid cells in vitro (Figure 5A‐5E).

In order to explore the roles of ERK1/2‐Cox2 signal in the EMT process induced by BPA exposure in Nthy‐*BRAF* ^V600E^ cells, we subsequently detected the expression of several key molecules in this pathway and observed a significantly increased p‐ERK and Cox2 levels (Figure 5F‐5H), suggesting the activation of ERK‐Cox2 pathway in Nthy‐*BRAF* ^V600E^ cells. Moreover, 10^‐7^ M BPA treatment significantly up‐regulated the p‐ERK and Cox2 levels in both of Nthy‐Vector and Nthy‐*BRAF* ^V600E^ cells, suggesting that the activation of ERK‐Cox2 was also induced by 10^‐7^ M BPA treatment. However, we did not observe a significant effect on ERK phosphorylation levels under the treatment of 3.33 × 10^‐4^ M BPA in the above two cells(Figure 5F and 5G). Remarkably, we observed a combinative effect of *BRAF* ^V600E^ mutation and 10^‐7^ M BPA exposure on the activation of the ERK‐Cox2 pathway (Figure 5F‐5H).

## 4 DISCUSSION

The *BRAF* ^V600E^ mutation as the most common genetic alteration in PTC and PTC‐derived undifferentiated thyroid carcinoma contributes to a constitutively active onco‐kinase^9^, ^20^ and is associated with extra‐thyroidal extension, metastases, recurrence, and mortality in PTC patients. A plethora of studies revealed that *BRAF* ^V600E^ controls a network of genes crucial in integrating and regulating the extracellular and intracellular signalling in thyroid cancer cells through the p‐MEK1/2 and p‐ERK1/2 pathway, which may be fundamental to trigger an abnormal cell differentiation/totipotency and shape/polarity, and contribute to tumour aggressiveness. Recently, an immunohistochemistry method with an anti‐*BRAF* ^V600E^ VE1, targeting the specific amino acid sequence (amino acids 596‐606), has been widely adopted to analyse *BRAF* ^V600E^ mutation status in routine pathological diagnosis.^17^ In the present study, the evaluation of the *BRAF* ^V600E^ mutation was identified by the immunohistochemistry stain with *BRAF* ^V600E^‐specific (VE1) in 26 out of 45 cases (57.8%), consistently with the previous reports, which demonstrated a higher incidence of *BRAF* ^V600E^ mutation in thyroid carcinoma.

Exposure to BPA via food consumption and environmental intakes in human beings has increased in recent years due to an increase in products based on epoxy resins and polycarbonate plastics. Our current study revealed that the BPA exposure level in PTC patients’ plasma (1.20 × 10^‐7^ to 3.50 × 10^‐7^ M) was slightly higher than the reported data ranging from 0.05 to 34.46 ng/mL (2.19 × 10^‐10^ to 1.51 × 10^‐7^ M),^21^ while the data were consistent with a study of 300 Korean women whose serum BPA content range was from 0 to 66.48 ng/mL (0 to 2.91 × 10^‐7^ M).^22^ The inconsistent exposure level of BPA may be due to the ethnic variation, sample size, survey accuracy and measurement mode. Although our results demonstrated that PTC patients carrying *BRAF* ^V600E^ mutation had higher plasma BPA levels than wild‐type ones according to statistical analysis, the real difference in biology significance needs to be further explored in the subsequent consideration.

Previous studies revealed that BPA exposure had the potential to induce metastasis and migration of cancer cells via activating several signalling pathways associated with the EMT program.^4^, ^16^ Our experiment also emphasized the importance of BPA exposure in the EMT process. Remarkably, BPA exposure accelerated the EMT process of PTC harbouring *BRAF* ^V600E^ mutation, which was evidenced by the significant down‐regulation of E‐cadherin and slight up‐regulation of N‐cadherin and MMP9.

A thyroid cell line Nthy‐ori 3‐1 cell harbouring wild‐type BRAF^23^ was used as an ideal cell model in the present study for further in vitro transfection. Our results demonstrated that the overexpressed *BRAF* ^V600E^ mutation could make the thyroid cells acquire some typical phenotype of mesenchymal cell, such as more elongated shape and less intercellular contacts, as reported the alteration in cell morphology during the EMT process mainly induced by the conversion of cytoskeletal proteins from keratin to vimentin.^24^ Notably, our in vitro data reminded 10^‐7^ M BPA exposure, a common human exposed dose could facilitate the process of EMT especially in PTC harbouring *BRAF* ^V600E^ mutation.

Overall less is known about the exact mechanisms by which BPA exposure could promote the EMT process of *BRAF* mutated thyroid cells. In light of previous research, BPA regulated Cox2‐mediated EMT in hemangioma cells,^25^ and *BRAF* ^V600E^‐induced EMT was dependent on the RAF‐MEK‐ERK signalling pathway,^26^, ^27^ our study introduced the ERK‐Cox2 signal pathway to explain the potential mechanism. As expected, our data further clarified whether BPA exposure or *BRAF* ^V600E^ induced EMT was mediated by activating the ERK‐Cox2 signalling pathway to enhance migration, invasion and clonality in thyroid cells, especially at 10^‐7^ M BPA exposure. However, 10^‐4^ M BPA treatment led to an inhibitory effect on the migration, invasion and clonality due to higher toxicity in thyroid cells. Undoubtedly, 10^‐7^ M BPA exposure will bring more serious health risks to PTC patients carrying *BRAF* ^V600E^ mutation. As 10^‐7^ M BPA exposure as a human exposed dose is of vital importance, our current study mainly focused on the health effects at such an exposed dose. Accordingly, it is necessary to suggest that patients suffering from thyroid disease should minimize the use of tableware and plastic products containing BPA in their daily lives.^28^

Taken together, this study has focused on the interaction between BPA exposure and *BRAF* ^V600E^ mutation on the EMT progress of PTC for the first time. Remarkably, BPA exposure at a human exposed dose can synergize with the *BRAF* ^V600E^ mutation to promote EMT phenotype formation of thyroid cells by activating the ERK‐Cox2 signalling pathway and further accelerate the migration/invasion of PTC. Although there are some limitations, our current study may contribute to understanding some shallow mechanisms underlying the combined effect of genetic mutation and endocrine‐disrupting chemicals exposure.

## CONFLICT OF INTEREST

No conflicts of interest exist regarding this study.

## AUTHOR CONTRIBUTION

Liuli Li: Conceptualization (equal); Data curation (equal); Formal analysis (lead); Investigation (lead); Software (equal); Validation (equal); Visualization (lead); Writing‐original draft (equal); Writing‐review & editing (lead). Hao Li: Conceptualization (equal); Data curation (equal); Formal analysis (equal); Investigation (equal); Validation (equal); Visualization (equal); Writing‐original draft (equal). Jun Zhang: Conceptualization (equal); Data curation (equal); Formal analysis (equal); Investigation (equal); Methodology (equal); Software (equal); Visualization (equal). Xin Gao: Investigation (supporting); Methodology (equal); Project administration (equal); Software (equal); Supervision (equal); Validation (equal). Hao Jin: Methodology (equal); Project administration (supporting); Resources (equal); Software (equal); Supervision (equal); Validation (equal). Renqi Liu: Investigation (supporting); Methodology (equal); Project administration (equal); Resources (equal); Software (equal); Supervision (equal); Validation (equal). Zhen Zhang: Investigation (equal); Methodology (equal); Project administration (supporting); Resources (supporting); Validation (equal); Visualization (equal). Xuan Zhang: Data curation (equal); Formal analysis (equal); Investigation (equal); Methodology (equal); Software (equal); Validation (equal). Xichang Wang: Investigation (equal); Methodology (equal); Software (equal); Visualization (equal). Peng Qu: Formal analysis (equal); Investigation (equal); Methodology (equal); Software (equal). Yuejiao Zhao: Conceptualization (equal); Funding acquisition (equal); Methodology (equal); Project administration (equal); Resources (equal); Supervision (equal); Writing‐review & editing (equal). Xiaobo Lu: Conceptualization (lead); Funding acquisition (lead); Project administration (lead); Resources (equal); Supervision (lead); Writing‐review & editing (lead).

## Supporting information

Supplementary Material
