# Supplementary material for: Bisphenol A at a human exposed level can promote epithelial‐mesenchymal transition in papillary thyroid carcinoma harbouring BRAF V600E mutation
Source: J Cell Mol Med. 2021 Jan 19;25(3):1739–49. doi: 10.1111/jcmm.16279 (PMC7875916; doi:10.1111/jcmm.16279)
Supplement: Supplementary file 1 — Supplementary Material [file JCMM-25-1739-s001.docx]

Supplementary Information

**Bisphenol A at a human exposed level can promote epithelial mesenchymal transition in papillary thyroid carcinoma** **harbouring *BRAF*^V600E^ mutation**

Liuli Li^1*^, Hao Li^1*^, Jun Zhang^2*^, Xin Gao^3^, Hao Jin^4^, Renqi Liu^4^, Zhen Zhang^4^, Xuan Zhang^1^, Xichang Wang^1^, Peng Qu^1^, Yuejiao Zhao^3#^ and Xiaobo Lu^1#^

1. Department of Toxicology, School of Public health, China Medical University, Shenyang, PR China.

2. Department of Oromaxillofacial–Head and Neck Surgery and Department of Oral and Maxillofacial Surgery, School of Stomatology, China Medical University, Shenyang, PR China.

3. Department of head and Neck Surgery, Cancer hospital of China Medical University**/**Liaoning Cancer hospital & Institute, Shenyang, PR China.

4. Jin Zhou Center for Disease Control and Prevention, Jinzhou, PR China.

*The first three authors Liuli Li, Hao Li, and Jun Zhang contributed to this work equally.

^#^Correspondence to: Dr. Xiaobo Lu and Dr. Yuejiao Zhao

**Table S1.** The primer information used for Quantitative Real-Time PCR

| **Gene** | **Primer Sequence 5’**-**3’** | **Product length** |
| --- | --- | --- |
| *BRAF* | AGACGGGACTCGAGTGATGATTGG (sense) | 170bp |
|  | TTGAAGGCTTGTAACTGCTGAGGTG (Antisense) |  |
| *E-cadherin* | ATTTTTCCCTCGACACCCGAT (sense) | 109bp |
|  | TCCCAGGCGTAGACCAAGA (Antisense) |  |
| *N-cadherin* | TGCGGTACAGTGTAACTGGG (sense) | 123bp |
|  | GAAACCGGGCTATCTGCTCG (Antisense) |  |
| *MMP-9* | ACGCACGACGTCTTCCAGTA (sense) | 94bp |
|  | CCACCTGGTTCAACTCACTCC (Antisense) |  |
| *GAPDH* | GGTATCGTGGAAGGACTC (sense) | 119bp |
|  | GGGATGATGTTCTGGAGAG (Antisense) |  |

**Table S2.** The basic information of study population

| **Variables** | **Healthy controls** | **PTC patients** | ***P* Value** |
| --- | --- | --- | --- |
|  | n(%) | n(%) |  |
| Total  Age(years) | 50(100) | 45(100) |  |
| <50 | 24(48.00) | 24(53.33) | 0.604 |
| ≥50 | 26(52.00) | 21(46.67) |  |
| Sex |  |  |  |
| Female | 39(78.00) | 36(80.00) | 0.811 |
| Male | 11(22.00) | 9(20.00) |  |
| Smoking |  |  |  |
| Yes | 12(24.00) | 16(35.56) | 0.217 |
| No | 38(76.00) | 29(64.44) |  |
| Drinking |  |  |  |
| Yes | 5(10.00) | 10(22.22) | 0.103 |
| No | 45(90.00) | 35(77.78) |  |


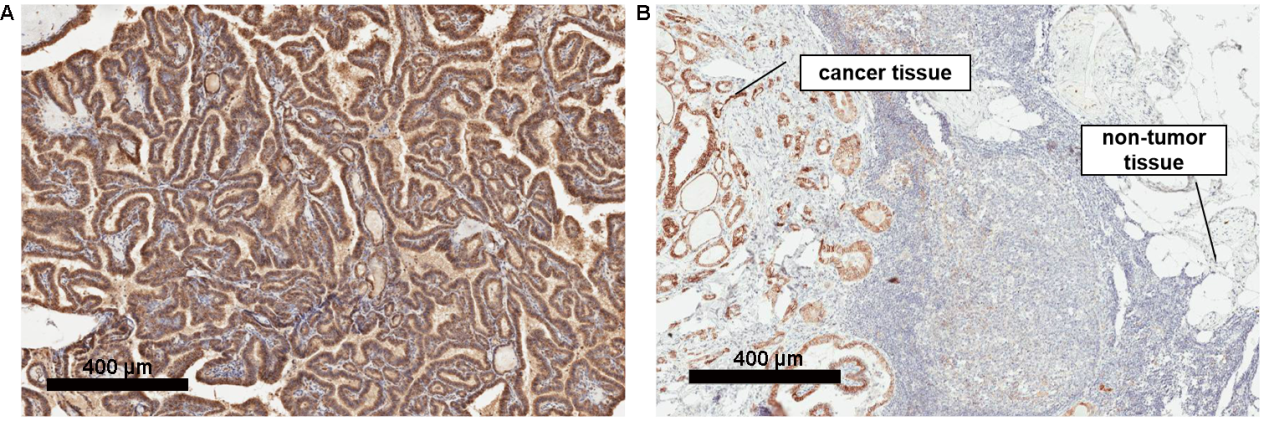


**Figure S1.** **Representative** **IHC staining of PTC with BRAF^V600E^ specific antibody**, VE1**.** (A) Representative for BRAF^V600E^ mutation (B) Representative for BRAF^V600E^ wild-type
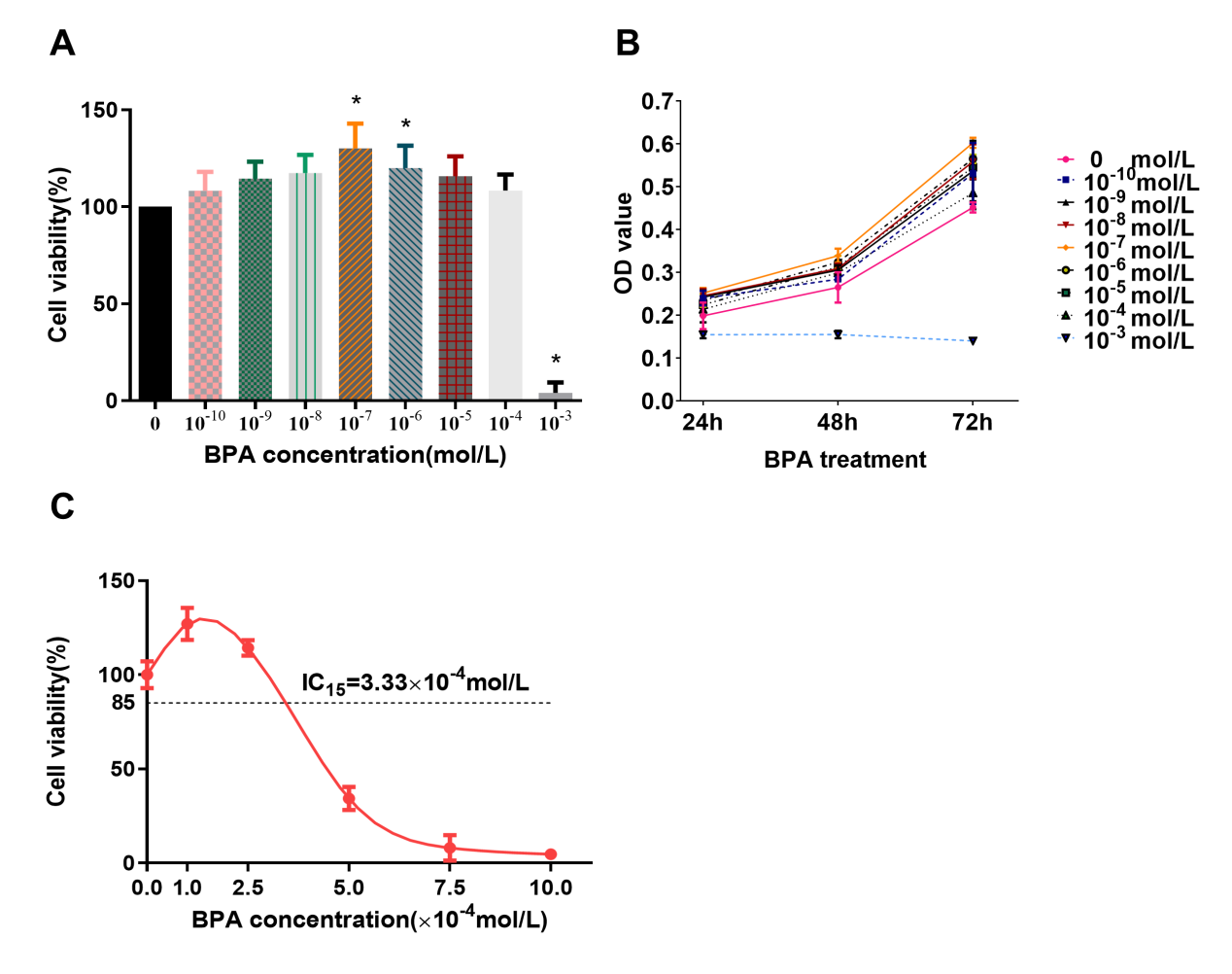


**Figure S2. Effect of different concentrations of BPA on cell viability.** (A) Cell proliferation experiment of Nthy-ori 3-1 cell exposed to 10^-10^ – 10^-4^ M BPA for 24h. **P*<0.05 *vs.* the control. (B) Cell proliferation experiment of Nthy-ori 3-1 cell exposed to 10^-10^ – 10^-4^ M BPA. (C) Growth curves of Nthy-ori 3-1 cells exposed to BPA ranging from 10^-4^ to 10^-3^ M.
